# The Value of HPA Axis Hormones as Biomarkers for Screening and Early Diagnosis of Postpartum Depression: Updated Information About Methodology

**DOI:** 10.3389/fendo.2022.916611

**Published:** 2022-07-12

**Authors:** Yujuan Chai, Qihang Li, Yang Wang, Enxiang Tao, Tetsuya Asakawa

**Affiliations:** ^1^ Department of Biomedical Engineering, Shenzhen University, Shenzhen, China; ^2^ Greater Bay Area International Institute for Innovation, Shenzhen University, Shenzhen, China; ^3^ Department of Neurology, The Eighth Affiliated Hospital, Sun Yat-Sen University, Shenzhen, China; ^4^ Institute of Neurology, The Third People’s Hospital of Shenzhen, Shenzhen, China; ^5^ Research Base of Traditional Chinese Medicine Syndrome, Fujian University of Traditional Chinese Medicine, Fuzhou, China

**Keywords:** axis hormones, adrenocorticotropic hormone (ACTH), behavioral assessment, cortisol, corticotrophin-releasing hormone (CRH), postpartum depression

## Abstract

Because of the high prevalence of postpartum depression (PPD) and the suffering involved, early diagnosis is urgent; however, current screening tools and diagnosis are inadequate. In addition to conventional methods such as the Edinburgh Postnatal Depression Scale and clinical interviews, several hormones in the hypothalamic–pituitary–adrenal (HPA) axis, such as corticotrophin-releasing hormone, adrenocorticotropic hormone, and cortisol, have been considered because of their critical roles in stress regulation in the mothers. The study designs are complicated, however, and so the effectiveness of these hormones as biomarkers for PPD is still controversial. Such inconsistency may have resulted from the variation in methodology between studies. The methodology problems in the investigation of PPD and HPA axis hormones have not been reported extensively. We therefore sought to summarize the methodological problems of studies published in the past decade, including the strengths and weaknesses of the examinations and the technological difficulties involved. Our findings suggest that (a) suitable samples and appropriate detection methods would reduce heterogeneity among trials; (b) the cutoff value of the scale test should be carefully selected for determining the performance of biomarker tests; (c) evaluation methods and criteria should be chosen with consideration of the tools feasible for use in local hospitals and population; and (d) the cost of diagnosis should be reduced. We hope that these findings provide insight for future investigations of HPA axis hormones as biomarkers for screening and early diagnosis of PPD.

## Introduction

Depression has become the largest contributor to global disability, causing approximately 800,000 suicide deaths per year (WHO,2017) (1). With a female-to-male risk ratio of 2:1, depression predominates among women, especially among those of childbearing age, who are vulnerable to maternal depression (2, 3). Postpartum depression (PPD) has a prevalence of 10%–15% worldwide (4), and approximately 50% of cases are undiagnosed and untreated (5). The complications of PPD include dysphoric mood, anxiety, sleep disturbance, suicidal thoughts, and neglect of the baby (6); the risk of future depression in the mother is 50%–62%, and the manifestations can cause long-term impairment in the development of offspring (7). Because PPD disrupts the psychological and mental health of the affected individual, early diagnosis or even screening testing is essential for the high-risk population. Early diagnosis and intervention may help reduce the suicide rate and improve quality of life for both mothers and infants.

Multiple approaches have been used to diagnose PPD. A well-designed interview according to the *Diagnostic and Statistical Manual of Mental Disorders, Fifth Edition*, is preferred (8), but in many cases, questionnaires are used, and the results are analyzed against other risk factors or biomarkers to increase their efficiency (9–11). However, it has been well documented that only using such subjective scales in a study might cause potential subjective bias (12, 13). In the clinical practice, the better solution is also application of some objective indices, along with these subjective assessments to achieve a better diagnosis (13). Hence, in addition to clinic interviews and self-rating scales, the profile of the endocrine system, particularly the hypothalamic–pituitary–adrenal (HPA) axis hormones, is taken into account (14, 15). Dysregulation of the HPA axis was frequently documented in people with major depression and PPD (16–18). Three important HPA axis hormones— corticotrophin-releasing hormone (CRH) (19), adrenocorticotropic hormone (ACTH), and cortisol—have been investigated intensively in prenatal and postnatal depression because the psychological changes in mothers may represent a unique mechanism of stress and parturition regulation (20, 21). Beginning in the 7th–10th weeks of pregnancy, the placenta secretes additional CRH (placental CRH), leading to dramatic increases in CRH, ACTH, and cortisol levels over the course of gestation (22, 23). The positive feedback loop of cortisol to placental CRH functions in addition to the negative feedback loop of cortisol to hypothalamus-generated CRH (24), serving as a biological timer that ends with parturition (19, 25). It has been suggested that placental CRH might initiate the labor process by stimulating the secretion of ACTH and boosting the cortisol level, which promotes delivery (23, 24, 26). Restoration of HPA axis hormones starts 2–4 days after parturition and may take up to 12 weeks to normalized (27). The desensitization of the HPA axis during this process and afterwards is postulated to be linked to PPD (28).

Despite the accumulated evidence of hypersecretion of stress hormones in major depression disorder and burnout syndrome, studies of HPA axis biomarkers in PPD have yielded inconsistent results (18, 29). In addition to hormone fluctuations, a large diversity of study designs might introduce uncertainty and difficulties in the investigation of prenatal and postnatal depressive disorder. Furthermore, because each research group was selected according to the investigators’ preferred scale or test thresholds, comparing subjective and objective methods is even harder (30). With the rapid development of *in vitro* diagnostic methods, accuracy and operation protocol for detection of hormones have been technologically improved, and new correlation and test results have been discovered (31, 32). With the need for early diagnosis of PPD, evaluation of a potential biomarker should also account for the compliance of patients during sampling, the operation of detection method and cost.

During the past decade, several comprehensive review articles were written about HPA axis hormones and their roles in prenatal and postnatal depression. For instance, Gelman et al. focused on the functional roles of HPA axis hormones and how they contributed to abnormalities in stress regulation during perinatal depression (33). Through a summary of existing literature and new clinical data, Glynn et al. proposed a model of how prenatal placental and HPA axis hormones can help predict the risk of postpartum depressive disorder (34). They provided evidence of increases in placental CRH, ACTH, and cortisol during pregnancy and clearly demonstrated the unique changes in the HPA axis hormone feedback loop. However, they did not mention methods of detecting the hormones and the sampling strategies. Likewise, in a mini-review, Dickens et al. summarized the normative changes in stress hormones in pregnant women and their critical roles in the development of depression and anxiety. However, they did not discuss the selection of specimens and diagnostic methods (35). The methods of stress hormone detection in PPD studies were analyzed in a systematic review by Garcia-Leal et al. (15), who focused on the heterogenicity of sample type (e.g., blood and saliva) and sampling time in studies of HPA axis hormones as biomarkers for PPD. However, they did not analyze the detection technique and the relationship between target hormones and detection tools, and failed to make a connection among the sampling strategy, test method and the outcomes of clinical trials. Of more importance, the sensitivity (cutoff) for the questionnaire and hormone tests should not be overlooked when analyzing the inconsistent conclusion of studies. Because of the requirements in clinical practice and research, these methodological issues must be summarized and analyzed; therefore, we reviewed this critical topic.

## Literature Search Strategy

**Figure 1:**
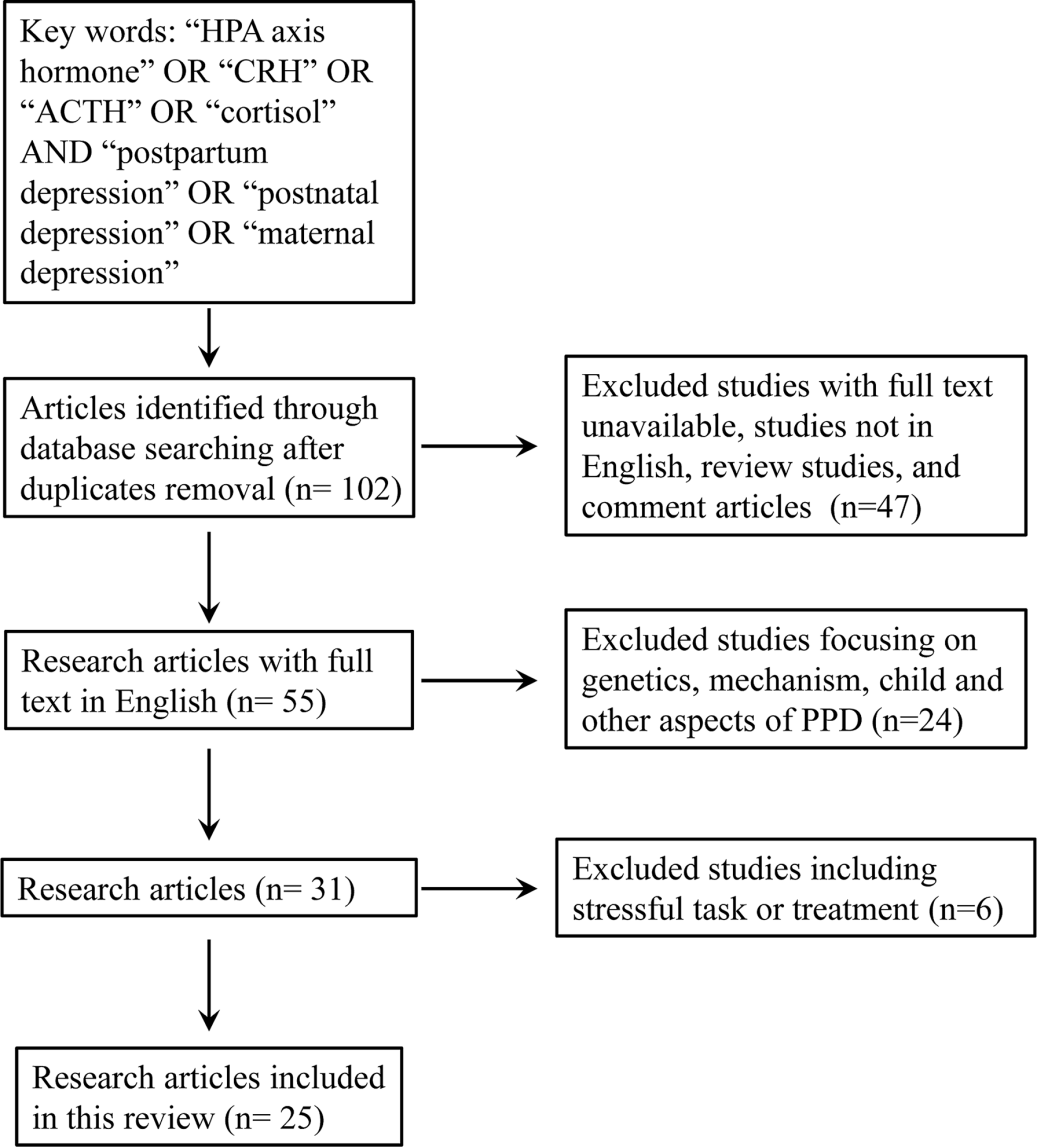
Flow chart of the search strategy and selection of the literature.

Our aim was to review the results of recent research (literature from 2010 to 2021) on CRH, ACTH, and cortisol, three well-characterized HPA axis hormones that can play a role in the diagnosis or prediction of PPD. We performed a comprehensive search using the following searching engines (PubMed, Google Scholar, Embase, and Web of Science). We used the keywords “HPA axis hormone” OR “CRH” OR “ACTH” OR “cortisol” AND “postpartum depression” OR “postnatal depression” OR “maternal depression”. The inclusion criteria were: research studies in human being published in English; available in full-text; studies involved the measurement of at least one of the three HPA axis hormones (CRH, ACTH and cortisol) in the pregnant or postpartum women; they should use at least one questionnaire to evaluate postpartum depression or depression symptom. The exclusion criteria were: animal study, review paper, studies not focusing on the evaluation method or prediction of PPD; studies regarding the children or fathers; studies involved stressful task or treatment before the collection of samples for hormone measurement. Finally, a total of 25 studies were selected for the further review (**Figure 1** and **Table 1**). We focused particularly on the detailed methodological issues involved. Moreover, the consistency and inconsistency between clinical trials is discussed with regard to conventional screening tools (questionnaires), sampling strategy, detection method, and target population. A total of We then provide an updated summary of the clinical/experimental application of these hormones and comments on the detection tools in terms of commercialization and operability for PPD evaluation. In light of these updated studies, suggestions for further reducing the heterogeneity among trials and increasing the applicability of clinical use will be given.

**Table 1:** CRH, ACTH and cortisol evaluation in the postpartum depression studies.

CRH-ACTH									
Sample type	Studies	Sample size	Biomarkers	Examination time	Sampling time	Methods	Scale tests	EDPS cut-off value	Results CRH-ACTH
Serum	Meltzer-Brody et al.,2011, USA (36)	1230	CRH	< gw20 + gw24-29	Morning	ELISA, LoD = 0.08 ng/mL, intra- and inter-assay CV < 5% and < 14%.	EPDS ppw12 + ppm12, CES-D in pregnancy	≥10, ≥13	CRH measures at less than gw20 and gw24-29 were inversely correlated with CES-D score at gw24-29. pCRH level was not correlated with EPDS score at ppw12 or ppm12.
	Cao et al., 2020, China (37)	185	CRH, 5-HT	gw20	NA	CRH: radioimmunoassay, 5-HT: ELISA	EPDS	>13	Serum level of both CRH and 5-HT was significantly correlated with EPDS score, and the area under curve of CRH/5-HT has a better specificity and sensitivity as indicator for PPD.
Plasma	Glynn et al.,2014, USA (38)	170	CRH,ACTH, Cortisol	gw15 + gw19 + gw25 + gw31 + gw36	Afternoon	Cortisol: ELISA, LoD = 0.25 ug/dL, intra-assay CV < 8%, ACTH: solid-phase two-site immunoradiometric assay, LoD = 1.0 pg/mL, intra- and inter-assay CV 4.4% and 10.8%, CRH: radioimmunoassay, LoD = 2.04 pg/mL, intra- and inter-assay CV 5%-15%,	EPDS, CES-D	> 13	Elevation of mid-gestation CRH and accelerated CRH trajectories were both associated with depression and EPDS score at 3-month but not 6-month postpartum.
	Hahn-Holbrook et al., 2013, USA (39)	210	CRH	gw19 + gw29 + gw37	NA	Radioimmunoassay, LoD = 10 pg/mL, intra- and inter-assay CV 3.7% and 5.2%	BDI	NA	Fewer depressive symptoms and more gradual increases in pCRH were predicted by family support significantly. Steeper increases in CRH predicted more PPD symptoms.
	Iliadis et al., 2016, Sweden (40)	535	CRH	gw17	NA	Radioimmunoassay, detection range 10-1280 pg/mL, intra- and inter-assay CV 1.7% and 3.0%	EPDS, SLE	≥12	High CRH levels in gw17 were significantly correlated with postpartum depressive at ppw6.
CFS	Zaconeta et al., 2015, Brazil (41)	129	CRH	During elective cesarean delivery, or underwent spinal anesthesia	NA	ELISA (EK-019-06 kit; PhoenixPhar maceuticals, licensed for CSF samples)	EPDS	≥13	CSF CRH level was not different between women with or without depressive symptoms both during pregnancy or in the postpartum period.
Plasma for CRH and ACTH, Serum cortisol	Labad et al., 2011, Spain (42)	132	CRH,ACTH, Cortisol	pph48	8:00 am-9:00 am	Serum cortisol: fluorescence polarization immunoassay, intra- and inter-assay CV 5% and 10%. Plasma ACTH: chemiluminescence, intra- and inter-assay CV 5% and 10%. Plasma CRH: radioimmunoassay intra- and inter-assay CV 5.5% and 10.2%	EPDS, STAI, DIGS, PRLES, FSSQ	≥9	No correlation between CRH or ln ACTH level and EPDS scores. A significant correlation was found between higher ACTH concentration and postpartum thoughts of harming the infant.
**Cortisol**									
**Sample type**	**Studies**	**Sample size**	**Biomarkers**	**Examination time**	**Sampling time**	**Methods**	**Scale tests**	**EDPS cut-off value**	**Results Cortisol**
Serum	Labad et al., 2011, Spain (42)	132	CRH,ACTH, Cortisol	pph48	8:00 am-9:00 am	Fluorescence polarization immunoassay, intra- and inter-assay CV 5% and 10%.	EPDS, STAI, DIGS, PRLES, FSSQ	≥9	Cortisol level had a significant positive correlation to ACTH level, but not CRH level pph48.No correlation between cortisol and EPDS scores at ppd2-3 and ppw8 were found.
	Saleh el et al., 2013, Egypt (43)	120	Cortisol, T3, estradiol	ppw1	NA	NA	EPDS, SCID-CV, IES, Fahmy and El-Sherbini’s Social Classification Scale	≥13	Greater postpartum drop in serum morning cortisol levels in the PPD group. Morning cortisol positively correlated with severity of depression
	Zhang et al., 2017, China (44)	255	Cortisol in mother and infant, prolactin in mother	mother: before delivery, infant: ppd3	NA	Radioimmunoassay, LoD = 2, intra- and inter-assay CV < 10% and 15%.	HAM-D, MAI, NBAS	NA	Maternal and neonatal serum cortisol in the depression group or whose mother belonged to the depression group were significantly higher than those in the normal group
	Gillespie et al., 2018, USA (45)	137	Cortisol	gw10-14 + gw20-24 + gw28-32w + ppw4-11	7:00 am and 1:00 pm	Chemiluminescence immunoassay, LoD = 0.2 μg/dL, intra- and inter-assay CVs of 7.1% and 7.9%	PSQI, PSS, CES-D, NUPDQ	NA	Primiparous women had higher cortisol level than multiparous woman during T2 and T3, along with a higher distress across pregnancy
	Adib-Rad et al., 2020, Iran (46)	80	Cortisol	ppm12	NA	ELISA	PSS, SCL-90-R	NA	Women with normal delivery had significantly higher cortisol level, PSS-14 and SCL-90 scores than recurrent pregnancy loss women
Plasma	Glynn et al.,2014, USA (38)	170	CRH,ACTH, Cortisol	gw15 + gw19 + gw25 + gw31 + gw36	Afternoon	ELISA, LoD = 0.25 ug/dL, intra-assay CV < 8%	EPDS, CES-D	> 13	No significant correlation was found for cortisol level during gestation and PPD symptoms at 3 month and 6 month measured by EPDS
Saliva	Ahn et al., 2015, USA (47)	119	Cortisol, IL-1β, IL-6, IL-8, IL-10, TNF-α, IFN-γ	gw32-36 + ppd7 + 14 + ppm1 + 2 + 3 + 6	awakening, 30 minutes later, 11:00 am, 4:00 pm, and 8:00 pm, at home	ELISA, LoD = 0.018 mg/dL. Intra- and inter-assay CV 4.3% and 5.2%	EPDS, PSS, Health Survey Questionnaire	> 10	No special correlation identified between cortisol level and PPD. Cortisol at 8am and 8:30am at ppm6 was higher in breast fed women compared to those who primarily bottle fed
	Corwin et al., 2015, USA (48)	152	Cortisol, IL-6, IL-8, IL-10, IFN-γ	gw32-36 + ppw1,2+ppm1,2,3,6	Awakening, 30 min, 11:00am, 4:00 pm, 8:00 pm, at home	ELISA, intra- and inter-assay CV 4.3% and 5.3%	EPDS, PSS	> 10	Higher cortisol AUC at ppd14 is significant predictors of PPD
	Pawluski et al., 2015, Sweden (49)	268 prenatal, 181 postpartum	Cortisol	gw36 + ppw6	20pm and 22pm, at home	ELISA, intra- and inter-assay CV 8% and 11%	EPDS, SLE	> 10	Higher evening cortisol in gw36 and postpartum in PPD women. Decrease in cortisol values from late pregnancy to postpartum
	Scheyer et al., 2016, USA (50)	100	Cortisol	<gw16 + gw17-30 + gw30-32 + ppm3	Awakening, 30, 45, 60 min after awakening;12:00 pm, 4:00 pm, 8:00 pm, at home	Time-resolved immunoassay with fluorescence detection, LoD = 0.43nM, intra- and inter-assay CV both <10 %.	EPDS, PSS	> 10	Higher stress level associated with flatter diurnal cortisol patterns in T3. No significantly associated between perceived stress and average cortisol output, AUC, or CAR at any time
	de Rezende et al., 2016, Brazil (51)	104	Cortisol	ppm6	Awakening,30 min, 3 h and 12 h after awaking, at home	Radioimmunoassay, LoD = 60 ng/dL, intra- and inter-assay CV 2.1% and 9.3%	EPDS, CES-D, PSS, HAM-D, SCID-CV, BAI, ESS	> 10	Relative increment in the CAR was significantly higher in healthy control than in euthymic postpartum women and depressive postpartum women. EPDS scores negatively correlated with CARi% in PPD patients
	García-Blanco et al., 2017, Spain (52)	148	Cortisol, α-amylase	gw38 + pph48 + ppm3	NA	Ultra-performance LC-MS, LoD = 0.05 nmol/L, intra- and inter-day CV 12% and 13%	BDI/SF, STAI, PSI/SF	NA	Cortisol levels increased sharply from 30 years of age at T3
	Luecken et al., 2019, USA (53)	322	Cortisol mother and infant	ppw12 + ppw2	Immediately before observational episode, at 0, 20, 40min after final episode, at home	NA	EPDS	NA	Prenatal depressive symptoms did not predict dyadic dysregulation, maternal cortisol, or infant cortisol. Maternal cortisol was only associated with infant cortisol
	Bublitz et al., 2019, USA (54)	197	Cortisol	salivary cortisol gw24 + gw30 + gw36 + ppd30	Awakening, 30min after awaking, bedtime	Immunoassay with time-resolved fluorescence detection	SES, QIDS	NA	SES was significantly associated with CAR at gw30 and weakly associated with evening cortisol at gw24 and gw36. Women with lower SES displayed flattening diurnal rhythm of cortisol across pregnancy
	Nazzari et al., 2020, Italy (55)	89	Cortisol, α-amylase, CRP, IL-6	gw34-36 + gw89 + pph52	30min after awaking and before bed	ELISA	EPDS, STAI-S	NA	Higher prenatal depressive symptoms were associated with lower cortisol levels at waking and 30 min after waking, postnatal cortisol has significant effect of time from delivery
Hair	Braig et al., 2016, Germany (56)	768	Cortisol	ppd0-3	NA	HPLC with MS/MS	TICS, PRAQ, HADS	NA	Hair cortisol were not correlated with self-reported chronic stress, anxiety, or depression
	Caparros-Gonzalez et al., 2017, Spain (57)	44	Cortisol	gw12, gw25, gw35, hair near scalp, <3 cm	NA	ELISA kit for saliva, LoD = 0.1ng/mL, intra- and inter-assay CV 2.7-4.3% and 4.4-6.3%	EPDS, PSS, SCL-90-R, GSI, PSDI, PDQ	≥ 10	Significant difference of hair cortisol levels between healthy and PPD participants in T1 and T3, significantly predicted EPDS scores
	Jahangard et al., 2019, Iran (58)	98	Cortisol, cortisone, progesterone, testosterone, DHEA	12week before delivery, ppw12, hair near scalp, <6 cm	NA	LC-MS/MS, LoD = 0.1pg/mg	EPDS, BDI	≥ 12	PPD is related to blunted hair cortisol both 12 weeks before and after delivery
	Stickel et al.,2020, Germany (59)	196	Cortisol, cortisone	ppd1–6 + ppw12	NA	SPE LC–MS	EPDS, SLESQ, MPAS, HAM-D	> 10	Decrease in cumulative hair cortisol from the T3 to ppw12 was significant only in the non-depressive group and adjustment disorder group but not depressive group
Urine	Shimizu et al., 2015, Japan (60)	54	Cortisol, adrenaline, noradrenaline	ppm1 + ppm4	before health check	NA	EPDS, General Health Questionnaire	≥9	No significant correlation was found between cortisol level and EPDS or GHQ score. Cortisol concentration positively correlated with adrenaline and noradrenaline at ppm1, adrenaline at ppm4

BAI, Beck Anxiety Inventory; BDI-SF, Beck Depression Inventory Short Form; CES-D, Center for Epidemiologic Studies Depression Scale; CRP, C reaction protein; CSF, cerebrospinal fluid; DHEA, dehydroepiandrosterone; DIGS, Diagnostic Interview for Genetic Studies adapted for postpartum depression; ELISA, Enzyme Linked Immunosorbent Assay; EPDS, Edinburgh Postnatal Depression Scale; ESS, Epworth Sleepiness Scale; FSSQ, Duke-UNC Functional Social Support Questionnaire; GSI, Global Severity Index; HADS, Hospital Anxiety and Depression Scale; gw, gestation week; HAM-D, Hamilton Rating Scale for Depression; HPLC, High Performance Liquid Chromatography; IES, Horowitz’s Impact of Event Scale; MAI, Maternal Attachment Inventory; MPAS, Maternal Postnatal Attachment Scale; MS, Mass spectrometry; NA, not available; NBAS, Neonatal Behavioral Assessment Scale; NUPDQ, Revised Prenatal Distress Questionnaire; pCRH, placenta CRH; PDQ, Prenatal Distress Questionnaire; ppd, day postpartum; pph, hour postpartum; ppm, month postpartum; PRAQ, Pregnancy Related Anxiety Questionnaire; PRLES, Paul Ramsey Life Experience Scale for life events; PSDI, Positive Symptom Distress Index; PSI/SF, Parenting Stress Index Short Form; PSQI, Pittsburgh Sleep Quality Index; PSS, Perceived Stress Scale; QIDS, Quick Inventory for Depressive Symptomatology; SCID-CV, Structured Clinical Interview for the DSM-IV clinical version; SCL-90-R, Symptom Checklist-90-Revised; SES, Socio-economic status; SLE, Stressful Life Event; SLESQ, Stressful Life Events Screening Questionnaire; STAI, State-Trait Anxiety Inventory; STAI-S, Subscale; T1, 1^st^ trimester of pregnancy; T2, 2^nd^ trimester of pregnancy; T3, 3^rd^ trimester of pregnancy; TICS, Trier Inventory for Chronic Stress.

## CRH Detection in PPD Studies

The peptide hormone CRH in the blood of pregnant women nearing delivery is secreted mainly by the placenta; ordinary production of CRH by the hippocampus is extremely low (61). As the principal regulator of the HPA axis and the key factor that controls physiological function during pregnancy and the onset of parturition, CRH has well-documented roles in the regulation of prenatal depression and PPD (19, 34, 62). We hypothesized that the gradual increase in CRH levels beginning in the second trimester, along with the sharp elevation during the short period of delivery, might play a role in the onset of PPD (27, 63).

### Detection Methods

The concentration of CRH in peripheral blood is extremely low in nonpregnant individuals and the samples should be handled cautiously if radioimmunoassay is applied. In PPD evaluation, investigators focused on the association of prenatal CRH level (including both hippocampal and placental CRH) at different stages of pregnancy and the onset of PPD. Unlike ACTH and cortisol, CRH is secreted in a pulsatile pattern, and sampling does not have to be performed at a specific time of day (64). However, if the three interacting stress hormones are studied together, a fixed sampling time should be set to make the comparison (42).

Because of the extremely low level of this biomarker, methods of sampling CRH are limited. Most studies involve CRH measurement assessed the secretion during pregnancy (testing pCRH), and immediate after delivery to predict the onset of postpartum depression. The mainstay assessments of CRH are plasma and serum levels, but cerebrospinal fluid is sometimes used. The combination of three HPA axis hormone was used in two other studies (38, 42): Labad et al. collected plasma in the mornings after delivery but found no strong association between scores of the Edinburgh Postnatal Depression Scale (EPDS) at 2 month postpartum and CRH level (42), Glynn et al. collected the sample in the afternoon from the 15th to 36th weeks of pregnancy and reported a predictive value of CRH concentration at mid gestation for postpartum depression at 3 month (38). In addition, three other studies supported the significant correlation between increased CRH concentration during the second or third trimester and depressive symptoms after delivery (37, 39, 40). Of note, the CRH level in all aforementioned studies were assessed with radioimmunoassay with highly sensitive and accurate: LoD 2-10 pg/mL, and intra- and inter-assay coefficient of variances (CVs) all below 15% (37–40).

Researchers in only two trials used enzyme-linked immunosorbent assay (ELISA) to detect CRH. One that involved 1230 participants revealed that serum CRH levels collected earlier than week 20 and between weeks 24 and 29 of pregnancy were negatively correlated with scores on the Center for Epidemiologic Studies Depression Scale (CES-D) during the second trimester but were not correlated with EPDS scores several months after delivery. With ELISA, the researchers were able to detect CRH in a minimum of 0.08 ng/mL of blood with intra- and inter-assay CVs of <5% and <14%, respectively (36). In the second study, Zaconeta et al. attempted to explore whether CRH from CSF could serve as a biomarker, but it failed to differentiate women with PPD from those without PPD (41). However, as pointed out by Hahn-Holbrook et al, only 6 women with postpartum depression was involved, making the statistical analysis unreliable. Another major concern was that impending surgery could contaminate the mood of participants and evaluation of depression on the delivery day might not serve as a good control (65).

### Evaluation of Methodology

Two techniques, radioimmunoassay and ELISA, were involved in the detection of CRH in blood or CSF in the aforementioned studies (39–41). Because the concentration of CRH is low, radioimmunoassay, which has very high sensitivity (approximately 100 times that of ELISA) is the method most commonly used to measure CRH (37, 66). The major disadvantage of radioimmunoassay is the complicated specimen preparation protocol, which requires low temperatures, long-time incubation, and radioactive element labeling. These complicated and risky steps limit the usage of radioimmunoassay. Currently, the method is rarely served in clinical application or a large-scale laboratory study (38, 66).

Moreover, discrepancies in operation procedures during CRH measurement, particularly the blood treatment steps, might introduce variability in measurement in this costly assay. An ELISA test kit is generally more commercially available and a safer option when the study population is large or the sample type is diverse (36, 41). However, the rationale for using ELISA is the convenience of the assay, rather than better performance, because the concentration of CRH during the first trimester and after delivery might be lower than the LoD of ELISA, and the results may therefore be uncertain (34).

## ACTH Detection in PPD Studies

ACTH circulates in blood and is secreted in a circadian rhythm similar to that of cortisol; its levels fluctuate between 4.1 and 51.4 pg/mL (32). It mediates the regulatory function of CRH and of downstream HPA axis hormones such as cortisol. ACTH is a key biomarker for the diagnosis of Cushing syndrome and adrenal tumors (32, 67). Because it has a short half-life in plasma (8–14 min) and the circulating concentration is low, ACTH has not been tested alone in PPD investment, but it is often studied along with CRH and cortisol (68).

### Detection Methods

ACTH measurement can be included in an experimental design in PPD studies if the other two stress hormones, CRH and cortisol, are measured in the same blood sample. Labad et al. using chemiluminescence assay (intra- and inter-assay CVs of 5% and 10%, respectively) for postpartum plasma ACTH assessment, found no correlation between plasma ACTH level and EPDS scores (42) but did find a significant relationship between higher plasma ACTH levels and mothers’ tendency to harm their infants, which is considered a reflection of psychological problems related to depression and anxiety (42). Both O’Keane et al. and Labad et al. assessed blood samples obtained during late gestation and after delivery. Glynn et al., in contrast, focused on the levels of stress hormones during weeks 15–36 of pregnancy but found no significant correlation between ACTH and either prenatal or postpartum depressive symptom (38). Glynn et al. used a solid-phase two-site radiometric assay with a good analytical sensitivity (1.0 pg/mL) and accuracy (intra- and inter-assay CVs of 4.4% and 10.8%, respectively) (38).

### Evaluation of Methodology

At present, chemiluminescence assay is the detection method mainly used in central laboratories in hospitals and in many bench studies (42, 69), whereas immunoradiometric assay is relatively convenient for two-site research (38). As mentioned, ACTH is difficult to evaluate alone as a potential biomarker in clinical research because of its very low concentrations in circulation, even during the third trimester. The stability of ACTH varies greatly in different test methods (31, 32). Accordingly, detection methods should be selected carefully in studies of ACTH.

## Cortisol Detection in PPD Studies

Hypercortisolism is a transient endocrine condition that is associated with a blunted HPA stress response in mothers. Studies of the correlation between cortisol level and PPD have demonstrated large inconsistencies in sample types, sampling times, and detection methods.

### Detection Methods

Because cortisol can be measured in different types of samples, and because of the numerous detection methods employed, selection of sampling strategy is crucial for the experimental design and the consistency of results. Specific detection methods are appropriate for different types of samples and are not interchangeable. With regard to *in vitro* diagnostic techniques, each method has a verified reference range and a suitable pretreatment protocol. The concentration of cortisol in blood samples is relatively high, with a well-defined 95% confidence interval in healthy populations that is determined by commercial test kits for chemiluminescence and ELISA. Some noninvasively obtained samples, such as saliva or hair, allow researchers to use multiple sampling times to investigate the complicated relationship between cortisol and PPD. However, the pretreatment of the sample and the performance of the detection methods are relatively challenging.

#### Invasive Sampling Methods

The invasive methods of sampling for cortisol include the collection of plasma or serum from peripheral blood. In 6 of the 20 studies listed in **Table 1**, peripheral blood was used because it allows simultaneous detection of other biomarkers of interest (31, 38, 42–46), particularly CRH and ACTH. Labad et al. and Glynn et al. independently found no significant correlation between early cortisol levels and EPDS scores 3 or 6 months after delivery (38, 42). Labad et al. used a fluorescence polarization immunoassay (intra- and inter-assay CVs of 5% and 10%, respectively) to measure cortisol in serum collected between 8:00 a.m. and 9:00 a.m. within 48 h of delivery (42); Glynn et al. used ELISA with a LoD of 0.25 µg/dL and an intra-assay CV of <8% for the detection of cortisol in plasma samples collected throughout pregnancy (38).

An Egyptian study of morning serum cortisol 1 week after delivery revealed a greater drop in cortisol level among depressed mothers than among nondepressed mothers and a positive correlation between severity of disease and cortisol concentration (43). Similarly, maternal and neonatal serum cortisol levels within 3 days after delivery were significantly higher in mothers with depression, which was diagnosed according to scores on the Hamilton Rating Scale for Depression. The cortisol concentration was measured with an iodine cortisol radioimmunoassay kit with a sensitivity of 2 ng/mL and intra- and inter-assay CVs of less than 10% and 15%, respectively (44). However, such radioimmunoassay with high sensitivity was rarely used in clinical application for cortisol measurement due to the abundance of this hormone as well as the inconvenient of operation. In a long-term observational study, Gillespie et al. measured the serum cortisol levels of participants in early, middle, and late pregnancy and after delivery and found that the correlation between cortisol level and PPD was highly related to parity; however, prenatal cortisol levels could not help predict the onset of PPD. The blood was sampled between 7:00 a.m. and 1:00 p.m. (4–11 weeks after delivery), and the assay was conducted with the solid-phase competitive chemiluminescence IMMULITE 1000 system (Siemens AG), with a sensitivity of 0.2 μg/dL and intra- and inter-assay CVs of 7.1% and 7.9%, respectively (45). Adib-Rad et al. conducted a case–control study of women 1 year after normal delivery and women 1 year after unexplained early pregnancy loss; they found that women with normal deliveries had higher stress or depression levels, determined by scores on the Perceived Stress Scale (14 items) and the Symptom Checklist–90, and higher cortisol levels than did women with unexplained early pregnancy loss (46). The serum samples in this study were obtained at 8:00 a.m., and cortisol was detected with a competitive enzyme immunoassay (IBL International Corp., Hamburg, Germany).

Invasive sampling for cortisol assessment—namely, collection of peripheral blood (serum or plasma)—was often conducted at a fixed time, as indicated in **Table 2**. Advantages of using peripheral blood are the well-studied blood matrix and low variation between individuals. In addition, peripheral blood collection is the most common sampling method in clinical examinations and is routine in hospitals; it is a basic technique involving a standard operating protocol and the same consumables; the development of most commercial automated machinery, along with the kits for testing hormone levels, was based on blood samples; and the process of sample preparation is very simple. However, this invasive sampling method has disadvantages: It is difficult to collect samples multiple times per day outside a clinic; some individuals may refuse such testing because of the inconvenience; and some individuals cannot tolerate the pain. Thus, it is preferable to use the minimum sampling times per day.

**Table 2:** Comparison of the invasive and non-invasive methods for detecting cortisol.

Items	Noninvasive	Invasive
Sample	Saliva, hair, and urine	Blood (plasma, serum) and CSF
Sampling methods	Swabs for saliva, hair near the scalp, and 24-h urine collection in a container	Peripheral blood collection and lumbar puncture
Clinical usage	Occasionally performed for endocrine disease	Blood samples are frequently obtained for endocrine disease, stress dysregulation, and infertility
Diagnostic method	ELISA for saliva and urine and LC-MS for hair	Chemiluminescence, ELISA, and lateral flow assay
Sampling conditions	Hospital or home, single or multiple time points, and variations in sample matrix and storage	Hospital, usually single time point per day, standard operation, consumables, and storage
Sample preparation	Easy for saliva, very complicated for hair	Easy and standardized
Detection time	Commonly 4–24 h for ELISA and >24 h for LC-MS	Commonly 1–2 h for chemiluminescence, 4–24 h for ELISA, and 10–15 min for lateral flow assay
Source of error	Self-sampling, storage, transportation, sample preparation, experimental operation, and quality of test kit	Calibration of equipment, experimental operation, and quality of test kit

CSF, cerebrospinal fluid; ELISA, enzyme-linked immunosorbent assay; LC-MS, liquid chromatography mass spectrometry.

#### Noninvasive Sampling Methods

Cortisol is detectable in multiple types of samples that can be obtained noninvasively, such as hair, sweat, and urine, during antepartum and postpartum periods. Of note, saliva cortisol level can reflect the diurnal rhythm of hormone secretion, hence often used for the construction of a dynamic curve in clinical studies. Ahn et al. found that cortisol levels in saliva collected at 4:00 p.m. 6 months after delivery were significantly and positively correlated with EPDS score (47). Corwin et al. reported a significant predictive value of higher cortisol AUC at postpartum day 14 for later depressive symptoms (48). Both studies used an ELISA test kit with a LoD of 0.018 mg/dL and intra- and inter-assay CVs of 4.3% and 5.2%, respectively, which is also the most common method used in detection of salivary cortisol (47, 48). Similar findings were reported by Pawluski et al., who collected saliva in the evening at week 36 of pregnancy and 6 weeks after delivery. The cortisol concentration measured with another ELISA test kit (intra- and inter-assay CVs of 8% and 11%, respectively) was significantly higher in women with self-reported depressive symptoms than in those without such symptoms and was positively correlated with EPDS score (49). In contrast, Nazzari et al. used ELISA to measure cortisol in saliva collected 52 h after delivery and found a negative association between prenatal depression and cortisol levels at waking and 30 min after waking (55). García-Blanco et al. focused on the fluctuation of cortisol levels in saliva and the emergence of mental impairment in participants of different ages. They found a sharp elevation in saliva cortisol concentration 3 months after delivery in mothers older than 30 years who had more severe symptoms of depression and stress. Saliva samples collected in their study were measured with ultrahigh-performance liquid chromatography mass spectrometry (LC-MS), with a LoD of 0.05 nmol/L and intra- and inter-assay CVs of 12% and 13%, respectively (52).

Investigation of the cortisol awakening response (CAR) 6 months after delivery also revealed a blunted increase in cortisol levels in depressed individuals in comparison with healthy controls. The relative CAR was negatively correlated with EPDS scores. A radioimmunoassay with a sensitivity of 60 ng/dL and intra- and inter-assay CVs of 2.1% and 9.3%, respectively, was used to determine saliva cortisol concentrations at three different times during the day of sampling (51). Self-collection of saliva samples also increases the feasibility of long-term monitoring of hormone fluctuation from the first trimester of pregnancy to several months after delivery. Using a time-resolved immunoassay with fluorescence detection (LoD of 0.43 nM, CV of <10%), Scheyer et al. found that PPD was associated with a lower level of CAR only in early pregnancy and mid-pregnancy (50). A flatter diurnal cortisol secretion pattern was observed in patients with worse depressive symptoms in the second and third trimesters and 3 months after delivery (50). Measurements of salivary cortisol at weeks 24, 30, and 36 of pregnancy and 1 month after delivery with time-resolved fluorescence detection immunoassay failed to demonstrate an association between PPD and cortisol level but did reveal a significant correlation between socioeconomic status and CAR at week 30 of pregnancy (54). In addition, saliva can be sampled to evaluate mother–infant dyadic dysregulation caused by the onset of psychological disorders. Luecken et al. collected saliva from both mothers and infants 12 weeks after delivery before and after observing dyadic interaction; they found that maternal and infant cortisol activity was significantly corelated but not associated with EPDS scores 24 weeks after delivery (53). As listed in **Table 1**, saliva cortisol could be detected with various methods without using complicated sample treatment. Most of the experimental designs involved a long-term or multiple-timepoint-monitoring of cortisol and depression levels.

Hair and urine samples are also safer and easier to obtain than is blood, but the extraction of cortisol from hair is complicated, and special technology such as LC-MS is required (56, 59). Braig et al. measured hair cortisol in women immediately after delivery, but it did not predict the development of depressive symptoms (56). In another study, the decrease in cumulative hair cortisol levels from the third trimester to 12 weeks after delivery was significant only in the healthy controls but not in the depressed participants (59). Based on a similar LC-MS of high sensitivity (0.1pg/mg), Jahangard et al. reported a predictive role of blunted hair cortisol in 12 weeks before and after labor to PPD. In this study, 6cm hair was taken near scalp, representing a long-term secretion of cortisol (58). Caparros-Gonzalez et al. used an ELISA test kit to measure cortisol in hair samples, which claimed to be technically feasible (LoD of 0.1ng/mL). Their data suggested that higher hair cortisol levels in the first and third trimesters could help predict EPDS scores with high reliability (57). Among the 20 articles about cortisol and PPD assessment, only one study, carried out in Japan, involved the use of urine samples; EPDS scores of 9 were indicative of depression. No correlation was found between urine cortisol at 1 or 4 months after delivery and EPDS score in that study (60).

In comparison with invasive sampling, as for blood, noninvasive sampling of saliva, hair, or urine facilitates participant recruitment and monitoring of cortisol level at multiple time points. However, it was mentioned in several articles that after women collected their own saliva with swabs, the collection tubes needed to be frozen until they could be delivered to the laboratory, or they had to be delivered to the laboratory immediately (47, 50, 51). The storage and pretreatment of hair sample were even more difficult to achieve, let alone the operation of LC-MS detection (56, 59). Because the processing of noninvasive samples collected for cortisol measurement is relatively complicated, this approach is not widely used in hospitals to evaluate PPD or other diseases.

### Evaluation of Methodology

The diversity of methodology selected for cortisol detection is due to the different “roles” of hormones in multiple types of tissues. For example, the hair cortisol is a retrospective measurement and serves as a “memory” of HPA axis functionality for weeks to months, whereas the 24h urine cortisol represents the secretion level in a day. The most frequently adopted peripheral blood sample for cortisol is often considered as a reflection of acute stimulation within 30-60mins or dysregulation of circadian rhythm and long-term stress response. The concentration of cortisol in these tissues is high enough for the sensitivity of multiple platforms compared with CRH and ACTH.

Among the commercially available *in vitro* diagnostic methods, automated multiplex methods, such as chemiluminescence immunoassay, are preferred in hospitals because of their convenience and robustness. In contrast, ELISA, which has less fixed costs and is more versatile, might be more suitable for experimental study in laboratories and with samples other than blood. As described in previous sections, serum samples allow simultaneous measurements of multiple biomarkers that are potentially associated with PPD, such as progesterone, estradiol, and ACTH (43, 69). The matrix of blood is relatively stable and well determined, and the development of most test reagents for hormones has been based on blood samples. Although peripheral blood can be obtained only by medical professionals, the collection process, consumables and sample preparation processes are commonly standardized, which helps prevent inconsistency in test outcomes among different examinations. During the past decade, saliva was used most frequently because patients could obtain samples themselves, which could effectively reduce the dropout rate in a long-term longitudinal study. Such noninvasive sampling methods are particularly invaluable in studies of the circadian rhythm of cortisol levels in patients with PPD. ELISA test kits designed for salivary cortisol were used in many bench studies (46, 47, 49, 57). Hair samples from near the scalp (approximately 1 cm in length) exhibit not transient levels of hormone secretion but a relative constant level over 1 month (57). Reliable however, among all mentioned detection methods, the sample preparation and operation of LC-MS specific for hair cortisol determination is the most complicated, necessitating careful handling by a well-trained expert.

## Assessments for Depression Screening

To improve the efficiency of screening, two types of self-evaluation tools were used in the previous studies: (a) scales designed particularly for pregnant women, such as the EPDS (10), the PPD Screening Scale (70), and the Pregnancy Risk Questionnaire (30); and (b) general depression scales, such as the CES-D (38), the Beck Depression Inventory (BDI) (52), and the Hamilton Rating Scale for Depression (44, 59). Such tools enabled evaluation during pregnancy of women at high risk for depression and those exhibiting early signs of depression (38, 45) and investigation of the psychological status of patients by documenting their socioeconomic status and sleep quality (51, 54).

### Effectiveness of the EPDS

The EPDS has been the most frequently used and best validated tool for evaluation of PPD for decades; the positive predictive value has been reported to fluctuate between 37% and 91% (10, 11, 71). There is no fixed cutoff EPDS score for depression, but a general acceptable threshold has been described for this convenient diagnosis tool (72). A recent meta-analysis documented that when the cutoff EPDS score varied between 9 and 13, the range of sensitivity of the scale correspondingly varied from 0.88–0.95 to 0.66–0.79, and the specificity also varied, from 0.71–0.78 to 0.90–0.95, depending on which reference standard interview was used (73). The screening power of the EPDS is widely accepted in both experimental and clinical usage, with minor adjustments of cutoff value according to a specific circumstance.

In the majority of the studies listed in **Table 1**, the EPDS was one of the tools used to screen for PPD, along with other generalized scales for depression or anxiety. The most frequently used cutoff values in the listed studies were 10 or higher (for cortisol studies) and 13 or higher (for CRH studies); scores above those values are generally considered to reflect “possible depression” and “probable depression” in PPD studies (74). The lowest cutoff value, 9, was used by two research groups. Shimizu et al. found no significant correlation between cortisol level and scale test scores (60), and Labad et al. found no correlation between cortisol, CRH, or ACTH concentration and EPDS scores (42). Of the five studies in which the cutoff value was 13 or higher, two demonstrated a significant correlation between blood CRH and postpartum depressive symptoms or EPDS score (37, 38), and one demonstrated a positive correlation between morning cortisol level and severity of depression (43). The remaining two studies revealed no relationship between CRH concentration and PPD; they were conducted with ELISA test kits for serum and CSF samples rather than radioimmunoassay, which is highly sensitive (36, 41). A higher cutoff value or specificity for the EPDS may be more suitable for screening for relatively severe symptoms or for more gradual onset of depression. Higher EPDS scores represent the most severe depression that patients experienced, which may have been caused by dysregulation of HPA axis hormones. Another reason for the inconsistency in findings of the studies might be the diagnostic tools used for stress hormone biomarkers. Both the EPDS and the examination method must have good sensitivity so that they can be used together to distinguish depressed patients from healthy ones. This issue discussed in detail in following sections.

### Other Scales and HPA Axis Hormones

In addition to the EPDS, a large variety of behavioral assessments have been used in studies of PPD, providing additional information about the psychological status of patients (**Table 1**). General depression questionnaires and anxiety questionnaires were commonly administered throughout pregnancy because the prediction and control of depression at earlier stages are always preferred and attempted (75, 76). On one hand, the neuroendocrine stress system naturally undergoes remarkable alterations during pregnancy and plays a key role in labor initiation (26, 77). Thus, HPA axis hormones after delivery should not be investigated separately from their historical trajectories (34, 78). On the other hand, HPA axis hormones are also found to be dysregulated in patients with anxiety, and so it is beneficial to identify PPD, anxiety, and, if present, even sleep disorder simultaneously (79, 80). As a result, the scales for these highly prevalent psychological disorders were administered together with the evaluation of HPA axis hormones over a long follow-up period in this target population.

The CES-D has been used in four studies among the articles listed in **Table 1**. One demonstrated that CRH levels at weeks 20 and 24–29 of pregnancy were inversely correlated with CES-D score (38). The Perceived Stress Scale, used in six studies, helped evaluate the onset of anxiety disorder (45–47, 50, 51, 57). Interestingly, all six studies involved the measurement of cortisol but not CRH or ACTH. The BDI was not frequently used, but it was the only questionnaire besides the EPDS that was used alone (39). As with the EPDS, a lower cutoff BDI score (9 or 10) could distinguish patients with mild symptoms, and a high cutoff score (18 or 19) was used to identify those with moderate to severe symptoms (9, 81).

In all studies of the role of HPA axis stress hormones in PPD, the aforementioned questionnaires were used for diagnosis in the participants, not only because each assessment generally took less than 15 min to finish but also because the sensitivity and specificity of the tests had been validated (30, 70, 72, 74). To overcome cultural differences and difficulties in understanding of these tests, the EPDS, BDI, and many other questionnaires have been translated into several languages and applied in different countries (37, 42, 60). However, even within a country or similar cultural background, different research groups may adopt different thresholds according to the aims of their studies. This may cause difficulties in comparing their conclusions.

## HPA Axis Hormones as Biomarkers for PPD

### Stress Response and the Role of HPA Axis Hormones During Pregnancy and Thereafter

Levels of HPA axis hormones, particularly CRH, ACTH, and cortisol, are dramatically altered during pregnancy, increasing sharply in the circulation of the mother and fetus; this scenario is similar to the situation in stress responses (22, 78). Moreover, the timing of parturition is determined by placental CRH, which serves as a “placental clock.” Through the regulation of ACTH, placental CRH may trigger a boost of downstream cortisol to initiate labor, which is an extreme perturbation of the HPA axis stress regulatory system (26, 38, 77). Fluctuation of neuroendocrine hormone levels, adjustment of family roles, and vulnerability to external stimulation might comprehensively contribute to the development of PPD. Therefore, assessment from different angles could possibly yield data that are useful for prediction, diagnosis and treatment.

### Reducing the Heterogeneity Among the Different Studies

Studies of CRH, ACTH, and cortisol since 2010 have been remarkably heterogeneous in terms of the sample type, sampling time and detection methods of the biomarkers, as well as their correlation with PPD. The reasons for this problem may be as follows: The sensitivity and specificity of both the scales and the method of detecting HPA axis hormones should be considered if these screening tools are used simultaneously. Problems with questionnaires, such as difficulty for patients to understand the questions, patients who fake symptoms, or patients who hide the real status of their health, generally do not affect the accuracy of biomarker tests. In contrast, because behavioral assessments do not involve sample collection methods, sampling timing, and specific techniques, the independence of the two approaches might help provide extra information about patients and help physicians better understand the onset of PPD.

The experimental designs of the studies listed in **Table 1** were diverse, particularly in the timing of both scale administration and hormone tests. For example, when psychological assessment was performed immediately before elective cesarean section, the participants were under stress other than depression (82). When the hormone concentration or secretion pattern is used to predict the onset of PPD, blood or tissue is usually collected during different stages of gestation. Nevertheless, the EPDS questionnaire was usually administered after the women gave birth; thus, a time gap existed between the two tests (37, 40). Scores on the depression or anxiety scales usually reflect the mental status of only the previous 1–2 weeks (10). This mismatch might further contribute to the inconsistencies of results in biomarker studies. It is therefore advantageous to arrange sampling times so as to counteract the potential influence of methodological differences.

Biomarker evaluation relies heavily on sample preparation and detection methods. The collection of samples for measuring CRH levels, which fluctuate in a pulsatile manner, may not be restricted to a particular time of the day, but sampling for ACTH and cortisol (in blood or saliva), which have ultradian rhythms, should be carefully timed (64, 83, 84). Cortisol concentration may fluctuate with time and age (85) and with seasons and external stimulation (86, 87), which results in different reference ranges and inconsistent conclusions. CRH and ACTH circulate at very low concentrations, and the measurement of ACTH requires careful handling and highly sensitive detection methods such as radioimmunoassay (66). As mentioned, two trials revealed no correlation between CRH concentration and PPD, but they were performed with an ELISA test kit rather than radioimmunoassay (36, 41). The inconsistency of conclusions cannot be attributed simply to the relatively lower sensitivity of ELISA, but the different detection techniques have made the comparison of results difficult. At present, an accurate, reliable, automated commercial method is indispensable. In addition, the experimental method should be selected in accordance with the characteristics of the hormones. For example, plasma ACTH can be maintained at 4°C or ambient temperature for 12 h with less than a 10% drop in detection if a Roche Cobas 6000 electro-chemiluminescence method is used instead of radioimmunoassay (31, 32). Cortisol is present at higher levels in blood and saliva and has better stability; the samples can be stored in a hospital laboratory and examined several commercial methods (26, 88, 89).

PPD studies are conducted worldwide, involving various races and populations. The fact that psychosocial factors vary across countries further contributes to the difficulty of comparing the results among different cultures (90). To improve the study quality, each study group should select their participants and the control group with carefully designed inclusion and exclusion criteria because HPA axis hormone secretion and stress responses may differ among races (91). Moreover, a background investigation is beneficial for the exclusion of uncertain cases, and publications of recent clinical trials may help researchers set reasonable thresholds for a local study.

### Advantages and Disadvantages

Although the correlations between HPA axis hormone levels and questionnaire results in PPD studies are controversial, investigations of the hormones as biomarkers have several advantageous. First, the concentration of a hormone is an objective reflection of the patients’ neuroendocrine status. The sampling protocol can be easily standardized and incorporated into the routine work of nurses and technicians. Second, multiple biomarkers can be simultaneously tested in one sample (47, 69). Other diseases such as diabetes, hypertension, and hyperglycemia commonly found during pregnancy can also be checked in parallel (92, 93). Third, prenatal screening of blood stress hormones can also be used to monitor stress regulation and metabolism, screen for prenatal depression or anxiety, and predict preterm labor and initiation of delivery (26, 94). The accumulation of data, in the long run, would further facilitate studies on the mechanism of placental biology and endocrinology.

The disadvantages of HPA axis hormone tests arise from the heterogeneity of studies and differences in experimental design, as previously discussed. Moreover, biomarker measurement alone provides no information regarding the background and socioeconomic status of participants, which are highly valuable in predicting the development of PPD (90, 95). In addition, the results reflect stress dysregulation and cannot predict the specific type of mental impairments (96, 97). So far, the sample size of most studies was relatively small due to the limitation in sampling or inconvenience of detection technique. It is difficult to address the role of additional influencing factors such as obesity in the dysregulation of HPA-axis hormones with limited data. Thus, the results of these studies must be comprehensively considered along with the results of questionnaires or symptoms before a final diagnosis can be established.

## Concluding Remarks

The early diagnosis and treatment of PPD are urgently needed because of its high prevalence and the suffering it causes. Fortunately, with the development of *in vitro* diagnostic techniques in the past decade, more testing methods and biomarkers have been verified and used for the screening of complicated diseases. Meanwhile, improvements in equipment and reagents have simplified both sample preparation and testing, while achieving higher sensitivity and accuracy. Although many investigators reported positive results, the inconsistency of the studies in terms of conclusion, experimental design, and methods is problematic. However, the combination of conventional behavioral assessments and hormone examinations may be an efficacious diagnostic strategy. Bioassays of hormones should be included in the regular screening for PPD in populations at high risk for depression, especially when pretreatment of test samples and the experimental operation are simple. In accordance with the insights obtained from the available literature, we have several suggestions for reducing inconsistency in future hormone examinations as a diagnostic tool for PPD:

- Researchers should collect more information about the dynamic change in levels of HPA axis hormones during pregnancy, delivery, and the postpartum period in large populations. The incorporation of stress hormone evaluation in a regular pregnancy (or postpartum) examination and selection of a matching detection method can help establish an appropriate threshold and reasonable references for future investigations.
- Because of the complicated nature of PPD and the limited methods of evaluation, cutoff values of scales should be carefully established to determine the performance of biomarker tests. More than one approach should be considered to improve the diagnosis or screening of PPD in populations at high risk for depression.
- With regard to behavioral assessments for PPD, insights about the behavioral tests for Parkinson’s disease—namely, principles of objectification, multipurpose use, and simplification in selecting and developing appropriate behavioral assessments (12, 13)—are also applicable for the development of behavioral assessments for patients with PPD, in which objectification is more important for the promotion of screening.
- In view of the prevalence of PPD and the urgent need for medical care, measures should be taken to reduce the cost of diagnosis, including labor costs, time consumption, and expenditures for the testing method. For example, an automated method can increase the scale of testing, and a point-of-care testing technique can reduce the time spent waiting for testing and results. Little labor input or training is required for these two diagnostic methods. In addition, development of an easier self-evaluation test that fits the culture and social status of local patients will also help.

A systematic experimental design to evaluate PPD should be based on the needs of patients and availability of medical resources, selection of a matching feasible method of detection in samples, establishment of a reliable threshold for the local population, and standardization of the stress hormone examination (**Table 3**). The objectification of the diagnosis/screening of a mental disorder is a huge challenge for physicians and researchers, as well as for affected patients. The development of novel assessments for PPD is urgently needed to promote the medical care of patients with PPD in the future and to improve the well-being of mothers and infants.

**Table 3:** Main experimental concerns and potential solutions involving the detection of HPA axis hormones.

Categories	Concerns	Potential solutions
Nature of the tools	Sensitivity, specificity, and reliability of the scoring system; cutoff time	Analyze the correlation according to different cutoff values (i.e., EPDS scores of 10–13), or address the correlation according to scores.
	Selection of different biomarkers	Consider the detection technique in clinical settings.
Experimental setting	Different sampling timing (antepartum and postpartum)	Combine the sampling points with regular antepartum and postpartum inspections according to local medical instructions.
	Variation in sample types	In addition to invasiveness and noninvasiveness, consider sample treatment procedure and availability of commercial tests.
	Variation in collection timing for periodic hormone	Consider the time of cortisol and ACTH sampling to eliminate the variation in levels caused by normal fluctuation caused by circadian rhythm.
Techniques	Preparation of different samples	Screening tool should be simple and easy to handle.
	Usage of an *in vitro* diagnostic technique	A safe, easily operated, commercialized method might help increase the reliability. Determine a reference range from a healthy population obtained with the selected method.
Target population	Exclusion criteria and background of participants	Historical health data and basic health status of patients should be specified in studies and analyzed together with the stress hormones.
	Socioeconomic and cultural differences	Verify whether questionnaires written in the local language are easy for target population to complete. For parallel comparison, results of other local studies can be used as references to establish cutoff values.

ACTH, adrenocorticotropic hormone; EPDS, Edinburgh Postnatal Depression Scale; HPA, hypothalamus–pituitary–adrenal.

## Author Contributions

YC and TA fetched the original ideas. YC, QL, YW, ET, and TA searched the literatures. YC and TA wrote the first draft, all the authors revised and approved the final version. TA supervised the study.

## Funding

This study was supported by Shenzhen Overseas Talent Program (827-000511), SZU Top Ranking Project (86000000210); SZU Start-up Grant (860-000002110806) and Guangdong Province Innovation Team “Intelligent Management and Interdisciplinary Innovation” (2021WCXTD002).

## Conflict of Interest

The authors declare that the research was conducted in the absence of any commercial or financial relationships that could be construed as a potential conflict of interest.

## Publisher’s Note

All claims expressed in this article are solely those of the authors and do not necessarily represent those of their affiliated organizations, or those of the publisher, the editors and the reviewers. Any product that may be evaluated in this article, or claim that may be made by its manufacturer, is not guaranteed or endorsed by the publisher.
